# Boosting EFL learners’ commitment and enjoyment in language learning through social networking: A literature review

**DOI:** 10.3389/fpsyg.2022.999586

**Published:** 2022-09-29

**Authors:** Bing Li

**Affiliations:** School of Foreign Languages, Jilin Institute of Chemical Technology, Jilin, China

**Keywords:** EFL learners, language education, social networking, well being, commitment, enjoyment

## Abstract

Social networking applications have been designed as innovative technologies used by the higher education section to enhance the acquisition of literacy skills, driving learners to engage in online learning platforms. Such tools such as social networking have also been proven to facilitate teaching and learning; therefore, educational programs and universities are increasingly making use of networking sites to form connections with students and to offer online instructional content. This trend has placed questions, regarding the effect of social media on language learning and its potential use as an effective instructional tool. The constructive role of enjoyment did not obtain equal academic consideration in the EFL learning milieu until Positive Psychology was presented to L2 education. Using social networking, users find online tasks appealing and experience the enjoyment that, in turn, impacts their behavioral intention to use them positively. Commitment is another factor that refers to the degree to which students are involved in learning enthusiastically. This construct concerns the extent to which students are loyal to learning, textbooks, and class etiquette. As it contributes to the quality of learning, it is considered a prerequisite for students’ academic success and educational attainment. Based on the role of the above-mentioned constructs in language learning, the present review tries to consider the role of social networking in boosting EFL students’ commitment and enjoyment. Consequently, some implications are presented for academic stakeholders in the quest of considering the role of social networking in language education.

## Introduction

Today, technology is used widely to integrate formal and informal education in the context of L2 learning and teaching (Khabir et al., 2022b). This allows the learners to benefit from the advantages associated with the effective application of technology both inside and outside the classroom (Botero et al., 2018). Accordingly, an increasing number of researchers have tried to shed light on the contributions of technology to the quality of L2 learning (e.g., Lee et al., 2016; Lai et al., 2018). The higher education institutes have adopted internet-based learning, i.e., virtual learning, in their different academic programs to enhance learners’ access and engagement while such technologies decrease costs and also bring about enjoyment and meaningful learning opportunities (Yang and Wu, 2021). Of much technological advancement, social networking Apps and their role in L2 learning have been in the spotlight that can enhance interactive contacts and the transmission of information with others in a virtual manner (Eid and Al-Jabri, 2016). Despite these advantages, the rapid use of these networks has raised concerns among many professionals, including experts, professors, and researchers regarding the efficacy and potential impact faced by students due to their obsession with online networking sites (Yapıcı and Hevedanlı, 2014). They believe that the incorporation of technologies into L2 learning courses in combination with traditional methods contributes to the general learning progress of the learners.

Given the development of cell phones and the widespread use of mobile devices, today, mobile social networks have emerged as the main channel of interaction among individuals (Zhou and Li, 2014). In recent years, the impact of mobile social networks on user behavior has caught the attention of researchers. Learners have chosen to use more than one account on social networking, which undermines their educational performance and influences their daily lives and sociability (Chen and Bryer, 2012). In the same vein, Chartrand (2012) maintains that social networking plays a role in increasing students’ enjoyment, motivation, and eagerness for L2 learning. Indeed, affected by Positive Psychology (PP), the scholars working in the field of Second Language Acquisition (SLA), as well as practitioners, have attached more importance to positive emotions than to negative emotional experiences (MacIntyre et al., 2016; Wang et al., 2021). Of multiple positive emotions (e.g., self-confidence, self-efficacy, esteem, enjoyment, etc.) experienced by L2 learners in different contexts (Dewaele and MacIntyre, 2016; Piniel and Albert, 2018), enjoyment has been in the spotlight given that it is deemed by L2 researchers as an essential component of achievement emotions that it is worthy of investigating as a result of its critical function in effective language learning (Piechurska-Kuciel, 2017). Li et al. (2018) characterize enjoyment as positive feelings emanating from going beyond homeostatic limits to fulfill something new or even innovative particularly, amid difficult tasks. Following the framework of a triple taxonomy of achievement emotions, one can categorize enjoyment as a positive, invigorating, and activity-oriented emotion, which has been realized to impact students’ academic achievement positively (Piniel and Albert, 2018).

Academically, the use of social networking enhances both the student’s motivation to learn, as well as the learning atmosphere in the classrooms. This is because such networks lead to the establishment of new relationships between learners and their teachers, or between peers (Wang, 2013). According to Cain and Policastri (2011), studies are more interesting or motivating when they are embedded in social networking as such networks enable the learners to feel and consider the presence of experts, in posts or videos. This ultimately makes the subjects more appealing to the learners that refer to their commitment that regards the level of physical and mental energy expended by the learners regarding their academic experience. In this context, several research investigations determined that the application of social networking as a teaching or learning tool can be expected to enhance learners’ commitment levels (Kaur et al., 2012; Al-Rahmi et al., 2014). The users of such a type of technology are likely to focus more on its hedonic use (e.g., enjoyment) than its educational use. A recent investigation concentrated on enjoyment as a determining factor refers to the role of social networking as a cause of enjoyment and pleasure (Sledgianowski and Kulviwat, 2009). Additionally, Csikszentmihalyi (2008) viewed enjoyment as an essential element of flow experiences whose main features are a high level of participation and engagement in a task, and which are conducive to L2 learning and development. Consequently, researchers need to examine how such constructs such as enjoyment may be impacted by the users of mobile social networks.

A review of the related literature shows that enjoyment has been deemed as a positive outcome, which may result in the people’s acceptance of specific information systems, a high level of satisfaction among them, a higher level of interaction with social network websites, and a constant focus on its real value (Sun et al., 2014). Likewise, one cannot ignore the significant role of enjoyment in learning. Accordingly, recent investigations have sought to clarify its theoretical implications for purpose of obtaining learning outcomes (Luczak and Kalbag, 2018). Some investigators have made a connection between learners’ enjoyment and their satisfaction or fun (Bashori et al., 2021).

Although Mobile-learning and social networking have received little attention in their language learning settings in previous decades and only a few schools care about them (Hockly, 2015; Kent et al., 2016), due to the Corona Pandemic, more attention is paid to it lately (Altam, 2020; Ying et al., 2021). Indeed, research has identified some features of social networking that contribute to the advancement of skills for communication between peers and school teachers or faculty members. They improve engagement, interactions, peer support, and commitment to educational tasks (Tiryakioglu and Erzurum, 2011). Seemingly, learners who cannot comply with new learning conditions and developments would encounter multiple challenges (challenges related to academic adjustment, social accommodation, emotional adaptations, and commitment to learning). Learners show more interest and commitment when they are valued, understood, and engaged in learning; consequently, commitment has drawn the attention of many researchers (Ahmad et al., 2017). Furthermore, there is a consensus among them to consider some types of emotions such as enjoyment as sociocultural products. They are formed and embedded in social interactions (Swain, 2013). Given the significant role of enjoyment and commitment in L2 learning, some studies should be carried out on how enjoyment and commitment are realized and evolved during learning. More specifically, the contributions of these two constructs have not been investigated concerning social networks. Consequently, this review seeks to deal with the application of social networking, with a focus on its effect on EFL learners’ enjoyment and commitment.

## Review of the literature

### Social networking

Social Networks have the power of changing how people engage in communication with each other (Villafuerte and Romero, 2017). As special forms of communication, they provide online communications manifested in programs such as Twitter, WhatsApp, and Instagram (Albashtawi and Al Bataineh, 2020). Accordingly, these technologies are deemed fast chatting ones that involve the provision of internet-based services. They are used to create personal profiles, post content, and interact with peers and teachers (Meng et al., 2017). As stated by Isisag (2012), new technologies and social media help to enhance the quality of foreign language learning (Mâţă, 2014). For example, social networking can improve the students’ learning process. Social media has emerged as an essential communication tool in that it enables researchers to share and distribute their information (Ajjan and Hartshorne, 2008; Mâţă, 2014). They can search for their required content such as information and documents, using online social networking platforms. The same Apps can also be used to interact with their instructors (e.g., WhatsApp, Twitter, and Instagram, among others; Aljaraideh and Al Bataineh, 2019; Albashtawi and Al Bataineh, 2020). In line with a study conducted by Golonka et al. (2014), technology provides teachers with an opportunity to enhance the quality of their teaching, using various rich sources. Technology impacts teaching and learning positively, improving learners’ motivation. Thanks to this technology, students can directly engage in situations where L2 is used; it makes it possible for them to engage in interaction and receive feedback from each other. Social networking is an internet-based platform that offers users multiple significant benefits like links, sharing opinions, times and information, speaking, interplay, and cooperation. Social networking is an online platform permitting users to have a half-or complete public profile accessible by society to speak, link, and talk with other mates and people (Boyd and Ellison, 2010). Social networking generally enhances discourse and interplay between people and teams throughout the world. In a review study, Trusov et al. (2009) concluded that generally, one could understand the notion of social networks easily. Such networks operate on the web, enabling users to create a profile identity and form subjective links among themselves. Liccardi et al. (2007) conducted a review of related studies and concluded that learners establish social and cultural links with each other. This makes it possible for them to share their learning experiences and engage in online interactions. Furthermore, universities today use online activities as an integral part of their daily programs. Such social networking provides students with opportunities to share new experiences, thoughts, skills, and advice (Junco, 2015). Moreover, they enable them to have online access to new information associated with their studies that can inspire their motivation and enjoyment.

### Enjoyment

Enjoyment is permanently defined as an initial/firm indicator of usage intention in hedonic information technologies, like social networking (Sledgianowski and Kulviwat, 2009). Constructive affections, like enjoyment, arrogance, and flow are taken into account as being effective in easing learning (MacIntyre and Gregersen, 2012). Enjoyment among the constructive affections is known as one of the most general constructive s that foreign language learners experience, which has gained rising consideration from scholars in the educational psychology area (Dewaele and MacIntyre, 2016; Li et al., 2018). Enjoyment refers to a feeling of consent and prize derived from tasks or succeeding in tasks (Ainley and Hidi, 2014). Enjoyment takes place when students understand themselves as skillful in doing an academic activity as well as understanding the learning procedure material (Mierzwa, 2019). The context of educational psychology usually describes enjoyment as a positive psychological mood derived from the attempts by individuals who go beyond themselves to complete a difficult or challenging thing (Csikszentmihalyi, 2008). Within the context of English language teaching, English enjoyment alludes to students’ willingness to learn EFL. Particularly, in the FL learning setting, experiencing enjoyment includes focusing, clarifying objectives, and quick feedback which assists students in constructing resources (Li et al., 2018). Each student with higher proficiency compared to other peers who finally gained greater degrees of competence in the goal language indicated a remarkably greater degree of enjoyment compared to their classmates (Dewaele and MacIntyre, 2016). The structure of enjoyment includes five classes, namely, behavioral, psychological, affective, expressional, and mental (Hagenauer and Hascher, 2014). As the name indicates, the emotional element of enjoyment concentrates on affections and specifically, on the feeling of satisfaction and joy experienced when learning procedures. Moreover, the psychological dimension of learning pertains to constructively assessing the condition. Therefore, enjoyment can be taken into account as the feeling of fulfillment that is generated when fulfilling a difficult, complicated activity that motivates investigation and builds passion (Ainley and Hidi, 2014).

In addition, the motivational dimension of enjoyment alludes to students’ capability to have a good feeling by motivating them, affectively and physically, to try higher future FL activities (Dewaele et al., 2018). Enjoyment alludes to the degree of involvement of Internet users in social networking because enjoyment is the component of assessing people’s decision to involve in social networking (Hsu and Lin, 2008). Enjoyment is an activity by which a person or team plays and people save pictures and videos in files and share them through one or more social networking (Eid and Al-Jabri, 2016). Nevertheless, enjoyment comprises two views: communicating with friends within the social network and assisting others (Moghavvemi et al., 2017). Internet users surely prefer tasks on social networking, since involvement increases enjoyment. Platforms for using social media offer an optimal chance for learning interplay and need higher effort in teaching entertainment (Hsu and Lin, 2008) that also cause their engagement and commitment.

### Commitment

Commitment refers to the subjective association between a worker and his or her institution (Fernet et al., 2016). This construct is concerned with emotional characteristics, including focused attention, feelings, and the adoption of constructive behaviors about particular matters. Commitment can be interpreted as a learner’s perseverance in higher education (Strauss and Volkwein, 2004), and academic commitment, which has recently been researched, is a multi-faceted concept (Viljoen, 2015). The majority of investigations in this area focus on the communication areas, with structural and academic commitment considered lately. Originally, a scholastic commitment was defined as the extent to which a learner is willing to invest in learning and academic activities (Viljoen, 2015). Commitment has to do with the affective variables associated with eagerness, faith, and the adoption of positive attitudes on particular things (Kim and Ok, 2009). As a sub-type of commitment, institutional commitment is concerned with general impressions, contentment, perceived sense of belonging, perspective on quality, and interest in a particular institution (Meyer and Allen, 2004). Having a strong commitment to the institution reinforces effective performance as learners obtain good marks and learners’ commitment is defined as an individual’s devotion to the university, allowing them to play a role in conveying and learning the culture, academic life, and campus facilities (Abduh et al., 2018).

### Related studies on social networking, commitment, and enjoyment

Lin and Lu (2011) pinpointed that people are interested in employing social networking services to experience enjoyment since they consider taking part in social media as a hobby beneficial for searching for evidence and sharing it with others in need. In addition, Zhang et al. (2021) examined the development of enjoyment by following students’ WeChat chats. They acknowledged some verbal languages as indicators of enjoyment such as a move to how learners used emojis and verbalized feelings with passion. There is another study conducted by Wang and Jiang (2021) in China to investigate learners’ enjoyment of virtual online learning during the COVID-19 pandemic, and the results indicated that learners had a great degree of enjoyment in an online learning milieu. Among social media platforms, Tiryakioglu and Erzurum (2011) indicated that Facebook has some features helping communication abilities with peers or faculty members, involvement, cooperation, peer support, and commitment to scholastic tasks. Moreover, it improves language education which has advantages both for learners and teachers in that it provides chances for engaging students in classes and developing their enthusiasm. Another study carried out by Resnik and Schallmoser (2019) probes the pedagogical structure focused on online cooperation, e-Tandem, which emphasizes two crucial modules for language education, including reciprocity and autonomy. Reciprocity is connected to students’ commitment to their own and their peers’ achievements. Al Ghazali (2020) tried to investigate the insights of students on social networking and the degree to which they take advantage of them to strengthen their linguistic presentation. The results display that social networking can contribute to the development of communication skills; however, these platforms do not work in cultivating writing and grammar. The learners stated that social networking assists them in their academic commitments and consequently leads to the progress of their communication and fluency.

## Conclusion and pedagogical implications

Through developing educational platforms used beyond the boundaries of the classroom and incorporating social networking sites into the instruction, the traditional ‘chalk-and-talk’ mode of teaching gives way to a novel educational concept of learning. Social networking is considered a subgroup of social media and allows students to engage in two-way communication and interactions with their peers *via* web-based and mobile technologies. Put it another way, such a type of technology facilitates the engagement in educational communication embedded in a milieu of an interactive dialog between learners. These networks are appealing to millions of users, transforming how they exchange and share information (Akbari et al., 2015). As a result, the relevant sites have turned into an integral part of the learning experience. Users have at their disposal a variety of options such as video, Music and post sharing, teleconferencing, online lecture, and video chats (Yapıcı and Hevedanlı, 2014; Akbari et al., 2015). Social networks provide a situation where learning occurs interactively, as many related investigations have revealed that learner interactions embedded in the context of social network communication influence learners positively (Sánchez et al., 2014; Chugh and Ruhi, 2018). One can view social networking sites as domains established within the framework of the new learning revolution that encourages their commitment to the computer industry that entails the direct role of the students. Indeed, the application of social media enhanced participants’ autonomous motivation directly, which may be attributable to their impression that such networks made L2 learning more enjoyable and interesting compared to traditional teaching methods (Huang et al., 2016). Finding English learning enjoyable, EFL learners lower their negative affective filter that contributes to facilitating L2 acquisition.

It can be concluded the application of technology and social networking concentrates on academic activities and make learning enjoyable. This helps the students to better understand the course contents as it increases the enjoyment of learning, making it an interactive experience (Baytak et al., 2011). Also, literature shows that enjoyment facilitates FL learners’ engagement or communication. This, in turn, increases their proficiency in processing L2 input and their mastery over L2 (Dewaele and MacIntyre, 2016). In particular, learners are interested in learning by doing, interacting, and discovering that it can promote learning and learners’ engagement. Learners’ commitment to their learning can be also reinforced through social networking. In such a context, these learners will be more eager to learn and the learners who find learning interesting will deeply engage in the learning process. Leaners’ commitment can be very helpful in enhancing their discussions, negotiation of meaning, and motivation. According to Reinhardt and Zander (2011), the application of social media in EFL classrooms speeds up L2 learning. Social networking Apps as new platforms make it possible for teachers to deliver authentic and student-generated L2 content, which improves learning, confidence, and motivation within the context of interactions and collaboration (Derakhshan and Hasanabbasi, 2015). Therefore, such platforms contribute to creating enjoyable and anxiety-free learning situations, which educators can use to make learning an enjoyable experience for students (McCarroll and Curran, 2013). As a result, those EFL learners who enjoy learning can become more interested in learning; develop in-depth thinking; gain a higher level of confidence; and improve their own performance which all brings about commitment (Dewaele and Alfawzan, 2018). This study is expected to have several implications in that it would provide educators and practitioners with some insights into the essential role of social networking in increasing learners’ enjoyment and commitment. Accordingly, officials in charge of developing education policies can draw on the review of the studies collected in this study to justify the incorporation of social networking in national educational programs, as this technology yields cost-effectiveness and supplements distance education that could enhance interactivity among learners. The national and local officials in charge of educational policies can benefit from this paper. For example, they can prioritize designing programs that make highly anxious learning situations more enjoyable. The review of the literature suggests that practitioners must lay the groundwork for an effective working and learning environment. This can be facilitated through using technology in general and social networking in particular as they increase learners’ enjoyment, perceptions of one’s efficacy, and commitment and consequently bring about enjoyment. This decreases learners’ anxiety and distress (Namaziandost et al., 2021), which in turn, leads to successful L2 learning.

Moreover, political officials in charge of educational policies as well as educators seek to lay the groundwork for the implementation of programs and instructional practices that transform the instruction, bringing about positive outcomes. Along the same line, educational programs try to reinforce learners’ achievements by incorporating technology such as social networking in such programs. Indeed, the design of game-like activities, interactive inquiries, and online assessments can be integrated into syllabuses through social networking to make learning fun. Educators and teachers can boost students’ commitment significantly by developing such fun-based problem-solving programs.

Furthermore, the conclusions reached by this review can have important implications for educators because they can play an essential role in creating an interesting, instructive classroom atmosphere. In the language context, teachers and educators provide all the necessary conditions for an atmosphere where EFL learners are encouraged to contribute to the activities to improve their learning. For instance, some tactics could be a good choice for teachers such as the use of various emojis and premeditated assistance in preparation, observation, and assessment. The teachers and educationists are encouraged to formulate modules that raise learners’ interest. For instance, they can integrate interactive programs so that learners’ interest is stimulated and their performance improves.

Moreover, the quality of teaching is at the center of attention which attaches great importance to the development of learners’ syllabus through creating a supportive classroom setting and facilitating connections using social networking with enjoyment. Teachers and educators can benefit from social networking as a teaching and learning tool. They can reinforce the traditional classroom environments given that they create ample opportunities to refine the curriculum through innovative, authentic, and/or flexible learning that boosts students’ enjoyment and commitment. L2 instructors need to know that modern conditions require doing away with the old style of teaching; instead, they should try modern teaching ways which require the inclusion of social networking sites to make learning effective and enjoyable for their learners’ commitment and enjoyment. Given the general impression that learners find social networking an enjoyable tool for communication, it is recommended that teachers benefit from such a technology in the L2 programs. This would promote L2 learners’ motivation and enjoyment and inspire life-long learning (Ebrahimzadeh and Alavi, 2016). Learning tasks reinforced by technology can increase motivation by inspiring the learners to take part in activities that are enjoyable and fulfilling and there is a consensus among researchers that collaborative activities done on social networks enhance learners’ commitment to the programs and reinforce their academic achievement (Huang et al., 2016). Teachers are advised to invest more time and resources in making learning enjoyable in L2 courses. One way to achieve this goal is the application of social networking, which has proved to be effective given that they create an atmosphere where L2 learners enjoy L2 learning. Thanks to the application of social networking, learners, teachers, and institutes can maintain their constant connections freely and conveniently. This would pave the way for the development of life-long learning, as well as the consolidation of learners’ commitment to continued independent learning.

Teachers play an important role in stimulating enjoyment, which influences L2 performance (Dewaele and Alfawzan, 2018); therefore, teachers need to undergo some training programs aimed at equipping them with the knowledge of how to implement activities embedded in social interactions through social networking. Such programs enhance both effectiveness and enjoyment. Above and beyond enjoyment, the role of commitment in language acquisition is pinpointed, and it is argued that both constructs are interrelated to better language presentation. Using social networking raised teachers’ involvement along with their motivation to develop materials as they took the role of the material developer which sequentially raised their satisfaction and interest in using it in teaching a language that leads to learners’ commitment.

## Suggestions for further research

Although this review focused on the effect of social networking on learners’ enjoyment and commitment, some limitations can be taken into account by the prospective studies. For example, given this paper aims to review the literature and did not have any treatment regarding the possible impact of social networking on learners’ enjoyment and commitment. This can be a concern for future studies. Similarly, studies in the future need to examine how the application of social media can also be best captured by qualitative research. Besides, more investigations must be conducted to compare the teachers’ perspectives on social networking with those of students in the context of L2 learning. More investigations must be conducted on the possible contribution of the learners’ commitment to enhancing educational processes. These studies should provide insights into the negative and positive emotions, perceptions, or behaviors that impact their commitment (Rodríguez-Izquierdo, 2020). Also, more studies can be done to consider the role of different models of instruction such as flipped classrooms (Khabir et al., 2022a) through social networking to examine the issue.

## Author contributions

The author confirms being the sole contributor of this work and has approved it for publication.

## Funding

This work was supported by Research on the Construction of College English Teachers’ Professional Community in Applied Undergraduate Colleges in the New Era (project number: JLICTMPC2020/029).

## Conflict of interest

The author declares that the research was conducted in the absence of any commercial or financial relationships that could be construed as a potential conflict of interest.

## Publisher’s note

All claims expressed in this article are solely those of the authors and do not necessarily represent those of their affiliated organizations, or those of the publisher, the editors and the reviewers. Any product that may be evaluated in this article, or claim that may be made by its manufacturer, is not guaranteed or endorsed by the publisher.

## References

[ref1] Abduh A. Rosmaladewi R. Basri M. (2018). Internationalisation awareness and commitment of Indonesian higher education. New Educ. Rev. 51, 162–172. doi: 10.15804/tner.2018.51.1.13

[ref2] Ahmad C. N. C. Shaharim S. A. Abdullah M. F. N. L. (2017). Teacher-student interactions, learning commitment, learning environment and their relationship with student learning comfort. J. Turk. Sci. Educ. 14, 57–72. doi: 10.12973/tused.10190a

[ref3] Ainley M. Hidi S. (2014). “Interest and enjoyment,” in International Handbook of Emotions in Education. eds. Pekrun R. Linnenbrink Garcia L. (New York, NY: Routledge), 205–227.

[ref4] Ajjan H. Hartshorne R. (2008). Investigating faculty decisions to adopt web 2.0 technologies: theory and empirical tests. Internet High. Educ. 11, 71–80. doi: 10.1016/j.iheduc.2008.05.002

[ref5] Akbari E. Pilot A. Simons P. R. J. (2015). Autonomy, competence, and relatedness in foreign language learning through Facebook. Comput. Hum. Behav. 48, 126–134. doi: 10.1016/j.chb.2015.01.036

[ref6] Al Ghazali F. (2020). Learners’ perceptions on using social networking sites to reinforce their linguistic performance. J. Lang. Ling. Stud. 16, 580–594. doi: 10.17263/jlls.759252

[ref7] Albashtawi A. Al Bataineh K. (2020). The effectiveness of google classroom among EFL students in Jordan: an innovative teaching and learning online platform. Int. J. Emerg. Technol. Learn. 15, 78–88. doi: 10.3991/ijet.v15i11.12865

[ref8] Aljaraideh Y. Al Bataineh K. (2019). Jordanian students’ barriers of utilizing online learning: A survey study. Int. Educ. Stud. 12, 99–108. doi: 10.5539/ies.v12n5p99

[ref9] Al-Rahmi W. M. Othman M. S. Musa M. A. (2014). The improvement of students ‘academic performance by using social media through collaborative learning in Malaysian higher education. Asian Soc. Sci. 10, 210–221. doi: 10.5539/ass.v10n8p210

[ref10] Altam S. (2020). Influence of social media on EFL Yemeni learners in Indian universities during Covid-19 pandemic. Ling. Culture Rev. 4, 35–47. doi: 10.37028/lingcure.v4n1.19

[ref12] Bashori M. van Hout R. Strik H. Cucchiarini C. (2021). Effects of ASR-based websites on EFL learners’ vocabulary, speaking anxiety, and language enjoyment. System 99:102496. doi: 10.1016/j.system.2021.102496

[ref13] Baytak A. Tarman B. Ayas C. (2011). Experiencing technology integration in education: children's perceptions. Int. Electron. J. Elementary Educ. 3, 139–151.

[ref14] Botero G. G. Questier F. Zhu C. (2018). Self-directed language learning in a mobile-assisted, out-of-class context: do students walk the talk? Comput. Assist. Lang. Learn. 32, 71–97. doi: 10.1080/09588221.2018.1485707

[ref15] Boyd D. M. Ellison N. B. (2010). Social network sites: definition, history, and scholarship. IEEE Eng. Manag. Rev. 38, 16–31. doi: 10.1109/EMR.2010.5559139

[ref16] Cain J. Policastri A. (2011). Instructional design and assessment. Using Facebook as an informal learning environment. Am. J. Pharm. Educ. 75, 207–215. doi: 10.5688/ajpe7510207, PMID: 22345726 PMC3279026

[ref17] Chartrand R. (2012). Social networking for language learners: creating meaningful output with web 2.0 tools. Knowledge management & e-learning: an. Int. J. 4, 97–101. doi: 10.34105/j.kmel.2012.04.009

[ref18] Chen B. Bryer T. (2012). Investigating instructional strategies for using social media in formal and informal learning. Int. Rev. Res. Open Distributed Learning 13, 87–104. doi: 10.19173/irrodl.v13i1.1027

[ref19] Chugh R. Ruhi U. (2018). Social media in higher education: a literature review of Facebook. Educ. Inf. Technol. 23, 605–616. doi: 10.1007/s10639-017-9621-2

[ref20] Csikszentmihalyi M. (2008). Flow: The Psychology of Optimal Experience. New York, NY: Harper Perennial.

[ref21] Derakhshan A. Hasanabbasi S. (2015). Social networks for language learning. Theory Pract. Lang. Stud. 5:1090. doi: 10.17507/tpls.0505.25

[ref22] Dewaele J. M. Alfawzan M. (2018). Does the effect of enjoyment outweigh that of anxiety in foreign language performance? Stud. Second Lang. Learn. Teach. 8, 21–45. doi: 10.14746/ssllt.2018.8.1.2

[ref23] Dewaele J. M. MacIntyre P. D. (2016). “Foreign language enjoyment and anxiety: the right and left feet of the language learner,” in Positive Psychology in SLA. eds. Gregersen T. MacIntyre P. D. Mercer S. (Bristol: Multilingual Matters), 215–236.

[ref24] Dewaele J. M. Witney J. Saito K. Dewaele L. (2018). Foreign language enjoyment and anxiety: the effect of teacher and learner variables. Lang. Teach. Res. 22, 676–697. doi: 10.1177/1362168817692161

[ref25] Ebrahimzadeh M. Alavi S. (2016). Motivating EFL students: E-learning enjoyment as a predictor of vocabulary learning through digital video games. Cogent Educ. 3, 1–13. doi: 10.1080/2331186X.2016.1255400

[ref26] Eid M. I. M. Al-Jabri I. M. (2016). Social networking, knowledge sharing, and student learning: the case of university students. Comput. Educ. 99, 14–27. doi: 10.1016/j.compedu.2016.04.007

[ref27] Fernet C. Trépanier S. G. Austin S. Levesque-Côté J. (2016). Committed, inspiring, and healthy teachers: how do school environment and motivational factors facilitate optimal functioning at career start? Teach. Teach. Educ. 59, 481–491. doi: 10.1016/j.tate.2016.07.019

[ref28] Golonka E. M. Bowles A. R. Frank V. M. Richardson D. L. Freynik S. (2014). Technologies for foreign language learning: a review of technology types and their effectiveness. Comput. Assist. Lang. Learn. 27, 70–105. doi: 10.1080/09588221.2012.700315

[ref29] Hagenauer G. Hascher T. (2014). Early adolescents’ enjoyment experienced in learning situations at school and its relation to student achievement. J. Educ. Train. Stud. 2, 20–30. doi: 10.11114/jets.v2i2.254

[ref30] Hockly N. (2015). Developments in online language learning. ELT J. 69, 308–313. doi: 10.1093/elt/ccv020

[ref31] Hsu C. L. Lin J. C. C. (2008). Acceptance of blog usage: the roles of technology acceptance, social influence and knowledge sharing motivation. Inf. Manag. 45, 65–74. doi: 10.1016/j.im.2007.11.001

[ref32] Huang C. S. Yang S. J. Chiang T. H. Su A. Y. (2016). Effects of situated mobile learning approach on learning motivation and performance of EFL students. J. Educ. Technol. Soc. 19, 263–278. Available at: https://search.proquest.com/docview/1768612513?pq-origsite=gscholar

[ref33] Isisag K. U. (2012). “The positive effects of integrating ICT in foreign language teaching,” in The International Conference ICT for Language Learning. 5th Edn. Florence, Italy: Pixel, 1–14. Available at: https://conference.pixelonline.net/conferences/ICT4LL2012/common/download/Paper_pdf/235-IBT107-FP-Isisag-ICT2012.pdf

[ref34] Junco R. (2015). Student class standing, Facebook use, and academic performance. J. Appl. Dev. Psychol. 36, 18–29. doi: 10.1016/j.appdev.2014.11.001

[ref35] Kaur S. Ganapathy M. Sidhu G. K. (2012). Designing learning elements using the multiliteracies approach in an ESL writing classroom. Southeast Asian J. Engl. Lang. Stud. 18, 119–134.

[ref36] Kent C. Laslo E. Rafaeli S. (2016). Interactivity in online discussions and learning outcomes. Comput. Educ. 97, 116–128. doi: 10.1016/j.compedu.2016.03.002

[ref37] Khabir M. Fazilatfar A. M. Razmi M. H. (2022a). Flipped presentation of IELTS Reading: impacts on grit, autonomy, and Reading achievement in an EFL context. Comput. Assisted Lang. Learn. 23, 178–197.

[ref38] Khabir M. Jabbari A. A. Razmi M. H. (2022b). Flipped presentation of authentic audio-visual material: impacts on intercultural sensitivity and intercultural effectiveness in an EFL context. Front. Psychol. 13:832862. doi: 10.3389/fpsyg.2022.832862, PMID: 35265019 PMC8899533

[ref39] Kim W. Ok C. (2009). The effects of relational benefits on customers' perception of favorable inequity, affective commitment, and repurchase intention in full-service restaurants. J. Hospitality Tourism Res. 33, 227–244. doi: 10.1177/1096348008329874

[ref40] Lai C. Hu X. Lyu B. (2018). Understanding the nature of learners’ out of-class language learning experience with technology. Comput. Assisted Lang. Learn. 31, 114–143. doi: 10.1080/09588221.2017.1391293

[ref41] Lee M. Pekrun R. Taxer J. L. Schutz P. A. Vogl E. Xie X. (2016). Teachers’ emotions and emotion management: integrating emotion regulation theory with emotional labor research. Soc. Psychol. Educ. 19, 843–863. doi: 10.1007/s11218-016-9359-5

[ref42] Li C. Jiang G. Dewaele J. M. (2018). Understanding Chinese high school students’ foreign language enjoyment: validation of the Chinese version of the foreign language enjoyment scale. System 76, 183–196. doi: 10.1016/j.system.2018.06.004

[ref43] Liccardi I. Ounnas A. Pau R. Massey E. Kinnunen P. Lewthwaite S. (2007). The role of social networks in students’ learning experiences. ACM Sigcse Bulletin 39, 224–237. doi: 10.1145/1345375.1345442

[ref44] Lin K. Lu H. (2011). Why people use social networking sites: an empirical study integrating network externalities and motivation theory. Comput. Hum. Behav. 27, 1152–1161. doi: 10.1016/j.chb.2010.12.009

[ref46] Luczak C. Kalbag A. (2018). The appropriateness and effectiveness of cross-aged peer mentoring in the learning environment. Int. J. Humanities Arts Social Sci. 4, 67–84. doi: 10.20469/ijhss.4.10003-2

[ref47] MacIntyre P. Gregersen T. (2012). Emotions that facilitate language learning: the positive-broadening power of the imagination. Stud. Second Lang. Learn. Teach. 2, 193–213. doi: 10.14746/ssllt.2012.2.2.4

[ref48] MacIntyre P. D. Gregersen T. Mercer S. (2016). Positive Psychology in SLA. Bristol: Multilingual Matters.

[ref49] Mâţă L. (2014). “Social media tools in initial teacher education,” in E-learning 2.0 Technologies and Web Applications in Higher Education. ed. Pelet J. E. (Hershey, PA: IGI Global), 129–154.

[ref50] McCarroll N. Curran K. (2013). Social networking in education. Int. J. Innovation Digital Econ. 4, 1–15. doi: 10.4018/jide.2013010101

[ref51] Meng J. Martinez L. Holmstrom A. Chung M. Cox J. (2017). Research on social networking sites and social support from 2004 to 2015: a narrative review and directions for future research. Cyberpsychol. Behav. Soc. Netw. 20, 44–51. doi: 10.1089/cyber.2016.0325, PMID: 28002686

[ref52] Meyer J. P. Allen N. J. (2004). TCM Employee Commitment Survey Academic Users Guide 2004. London, ON: The University of Western Ontario, Department of Psychology.

[ref53] Mierzwa E. (2019). Foreign language learning and teaching enjoyment: teachers’ perspectives. J. Educ. Culture Soc. 10, 170–188. doi: 10.15503/jecs20192.170.188

[ref54] Moghavvemi S. Sharabati M. Paramanathan T. Rahin N. M. (2017). The impact of perceived enjoyment, perceived reciprocal benefits and knowledge power on students’ knowledge sharing through Facebook. Int. J. Manage. Educ. 15, 1–12. doi: 10.1016/j.ijme.2016.11.002

[ref55] Namaziandost E. Razmi M. H. Hernández R. M. Ocaña-Fernández Y. Khabir M. (2021). Synchronous CMC text chat versus synchronous CMC voice chat: impacts on EFL learners’ oral proficiency and anxiety. J. Res. Technol. Educ. 1-18, 1–18. doi: 10.1080/15391523.2021.1906362

[ref56] Piechurska-Kuciel E. (2017). “L2 or L3? Foreign language enjoyment and proficiency,” in Multiculturalism, Multilingualism and the Self. Studies in Linguistics and Language Learning. eds. Gabrys-Barker D. Galajda D. Wojtaszek A. Zakrajewski P. (Cham: Springer), 97–111.

[ref57] Piniel K. Albert Á. (2018). Advanced learners’ foreign language-related emotions across the four skills. Stud. Second Lang. Learn. Teach. 8, 127–147. doi: 10.14746/ssllt.2018.8.1.6

[ref58] Reinhardt J. Zander V. (2011). Social networking in an intensive English program classroom: a language socialization perspective. CALICO J. 28, 326–344. doi: 10.11139/cj.28.2.326-344

[ref59] Resnik P. Schallmoser C. (2019). Enjoyment as a key to success? Links between e-tandem language learning and tertiary students’ foreign language enjoyment. Stud. Second Lang. Learn. Teach. 9, 541–564. doi: 10.14746/ssllt.2019.9.3.6

[ref60] Rodríguez-Izquierdo R. M. (2020). Service learning and academic commitment in higher education. J. Psychodidactics 25, 45–51. doi: 10.1016/j.psicoe.2019.09.00

[ref61] Sánchez R. A. Cortijo V. Javed U. (2014). Students’ perceptions of Facebook for academic purposes. Comput. Educ. 70, 138–149. doi: 10.1016/j.compedu.2013.08.012

[ref62] Sledgianowski D. Kulviwat S. (2009). Using social network sites: the effects of playfulness, critical mass, and trust in a hedonic context. J. Comput. Inf. Syst. 49, 74–83. doi: 10.1080/08874417.2009.11645342

[ref63] Strauss L. C. Volkwein J. F. (2004). Predictors of student commitment at two-year and four-year institutions. J. High. Educ. 75, 203–227. doi: 10.1353/jhe.2004.0007

[ref64] Sun Y. Liu L. Peng X. Dong Y. Barnes S. (2014). Understanding Chinese users ‘continuance intention toward online social networks: an integrative theoretical model. Electron. Mark. 24, 57–66. doi: 10.1007/s12525-013-0131-9

[ref65] Swain M. (2013). The inseparability of cognition and emotion in second language learning. Lang. Teach. 46, 195–207. doi: 10.1017/S0261444811000486

[ref66] Tiryakioglu F. Erzurum F. (2011). Use of social networks as an educational tool. Contemporary. Educ. Technol. 2, 135–150. doi: 10.30935/cedtech/6048

[ref67] Trusov M. Bucklin R. Pauwels K. (2009). Effects of word-of-mouth versus traditional marketing: findings from an internet social networking site. J. Mark. 73, 90–102. doi: 10.1509/jmkg.73.5.90

[ref68] Viljoen B. (2015). The Relationship Between Academic Commitment and Resilience for Education Students at the University of Pretoria. doctoral dissertation. South Africa: University of Pretoria.

[ref69] Villafuerte J. Romero A. (2017). Learners’ attitudes toward foreign language practice on social network sites. J. Educ. Learn. 6, 145–158. doi: 10.5539/jel.v6n4p145

[ref70] Wang X. (2013). Why students choose STEM majors: motivation, high school learning, and postsecondary context of support. Am. Educ. Res. J. 50, 1081–1121. doi: 10.3102/0002831213488622

[ref71] Wang Y. L. Derakhshan A. Zhang L. J. (2021). Researching and practicing positive psychology in second/foreign language learning and teaching: the past, current status and future directions. Front. Psychol. 12:731721. doi: 10.3389/fpsyg.2021.731721, PMID: 34489835 PMC8417049

[ref72] Wang Q. Jiang Y. (2021). A positive psychology perspective on positive emotion and foreign language enjoyment among Chinese as a second language learners attending virtual online classes in the emergency remote teaching context amid the COVID-19 pandemic. Front. Psychol. 12:798650. doi: 10.3389/fpsyg.2021.798650, PMID: 35095684 PMC8790507

[ref73] Yang Y. Wu J. (2021). Investigation and analysis on the fragmented learning status of college students under the background of online learning. J. Adv. Educ. Philos. 5, 66–69. doi: 10.36348/jaep.2021.v05i03.001

[ref74] Yapıcı I. U. Hevedanlı M. (2014). Educational use of social networks: Facebook case study. Eur. J. Res. Educ. 2, 16–21.

[ref75] Ying Y. H. Siang W. E. W. Mohamad M. (2021). The challenges of learning English skills and the integration of social media and video conferencing tools to help ESL learners coping with the challenges during COVID-19 pandemic: a literature review. Creat. Educ. 12, 1503–1516. doi: 10.4236/ce.2021.127115

[ref76] Zhang Z. Liu T. Lee C. B. (2021). Language learners’ enjoyment and emotion regulation in online collaborative learning. System 98:102478. doi: 10.1016/j.system.2021.10247

[ref77] Zhou T. Li H. (2014). Understanding mobile SNS continuance usage in China from the perspectives of social influence and privacy concern. Comput. Hum. Behav. 37, 283–289. doi: 10.1016/j.chb.2014.05.008

